# Footprints as Morphometric Evidence for Somatic Prediction and Body Proportion Reconstruction in Forensic Medicine

**DOI:** 10.3390/diagnostics16132114

**Published:** 2026-07-06

**Authors:** Fatma Çam Aygün, Serdar Babacan, Tuğçe Koca Yavuz, Kenan Kaya

**Affiliations:** 1 Adana Forensic Medicine Group Presidency, Council of Forensic Medicine, Ministry of Justice, Seyhan, 01060 Adana, Türkiye; drfatmacam@gmail.com; 2 Department of Anatomy, Faculty of Medicine, Bursa Uludağ University, Nilüfer, 16059 Bursa, Türkiye; sbc010777@gmail.com; 3 Council of Forensic Medicine, Ministry of Justice, Bahçelievler, 34196 Istanbul, Türkiye; 4 Department of Forensic Medicine, Faculty of Medicine, Çukurova University, Sarıçam, 01330 Adana, Türkiye; k_kaya_71@hotmail.com

**Keywords:** forensic medicine, footprint analysis, sexual dimorphism, discriminant function, regression model, biometric identification

## Abstract

**Background/Objectives**: This study aimed to apply morphometric and multivariate analytical techniques to footprint evidence for forensic identity determination, focusing on sex estimation and the reconstruction of body proportions as components of the biological profile. By integrating detailed footprint metrics with body measurements, the research sought to develop discriminant and regression models to evaluate the predictive value of footprint metrics for sex estimation and selected somatic dimensions. **Methods**: Static bilateral footprints were obtained using charcoal powder impressions and digitized using ImageJ. Eleven footprint parameters (F1–F11) and eleven body measurements (B1–B11) were recorded. Sex-based differences were examined using appropriate parametric or non-parametric tests with effect sizes. Sex estimation was evaluated using discriminant function analysis and internally validated using leave-one-out and stratified 10-fold cross-validation. Regression models for stature and body dimension estimation were assessed with multicollinearity diagnostics and repeated 10-fold cross-validation, including RMSE, MAE, and cross-validated R^2^. **Results**: The apparent discriminant classification accuracies were 74.0% for the right foot and 71.0% for the left foot. After internal validation, classification performance decreased to approximately 64–67%, indicating moderate discriminative ability. Reduced regression models showed the most stable validated performance for stature and arm span, although cross-validated R^2^ values remained weak. **Conclusions**: Static footprint morphometry may provide supportive information for sex estimation and selected somatic dimensions in this Turkish adult sample. However, the validated performance indicates that these models should be interpreted as ancillary and exploratory tools rather than standalone forensic identification methods.

## 1. Introduction

Forensic anthropology is a scientific discipline concerned with establishing individual identity from human anatomical structures, constructing the biological profile of remains, and contributing to the identification of living individuals. It estimates biological variables—primarily age, sex, stature, and ancestry—and evaluates these characteristics as evidence in forensic cases, including the analysis of findings recovered from crime scenes to support criminal investigations [1,2,3].

Fingerprint, dental, and DNA analyses are considered “primary” identification methods and are widely used for positive identification due to their high accuracy, particularly in cases of natural disasters, major accidents, and mass fatalities. However, the high cost and practical limitations of DNA analysis mean it cannot be applied in every case. As a result, the determination of secondary characteristics—such as age, sex, stature, and ancestry—is often preferred to narrow the pool of potential DNA matches and guide investigators. Secondary methods, including visual recognition, personal belongings, and photographic comparison, serve as supportive tools. While the effectiveness of primary methods depends on the condition of the remains and the availability of records, they remain essential for establishing identity with a high degree of certainty [4,5].

Footprints create a specific “pattern” unique to each individual, and this pattern can be two-dimensional due to a harder surface or three-dimensional due to a softer surface [6]. Bare footprints left at crime scenes constitute important physical evidence, reflecting the contact points and individual characteristics of the foot—a complex, functional part of human anatomy. They are a significant subject of study across forensic science, biomechanics, anthropology, and paleontology, where they are used to identify individuals, analyze gait patterns, and reconstruct movements during incidents. In investigations of theft, robbery, homicide, assault, and traffic accidents, bare footprints provide valuable clues that aid in case resolution and suspect identification [7,8].

The ratios between different parts of the human body are called “proportions”; the set of rules defining these ratios is called ‘canon’ in science and art. In artistic anatomy, the unit of measurement used to regulate body proportions or ensure the harmony of structural elements is called a “module.” Height is considered the fundamental element of a figure, and the widths of the head, torso, and extremities, as well as the hips, chest, and shoulders, are evaluated in relation to each other and to the total height [9].

Although numerous studies have examined footprints for sex estimation and stature prediction, footprint morphology varies among populations owing to genetic, environmental, nutritional, and lifestyle-related factors [10]. Predictive equations developed in one population may therefore not be directly applicable to another. Moreover, most previous studies have focused on sex estimation and stature reconstruction, whereas the relationship between footprint morphology and other body proportions has received comparatively little attention. Population-specific studies are consequently needed to establish reliable forensic reference data and improve the applicability of footprint-based identification methods.

The present study aims to apply analytical techniques to footprint morphometry as a reliable source of biometric data for forensic identity determination. Specifically, in a Turkish adult sample, the study applies multivariate discriminant function analysis to estimate sex from footprint characteristics and multiple linear regression to reconstruct body proportions, thereby contributing to biological profiling in cases where primary identification methods are unavailable. Furthermore, linear regression equations will be developed from footprint measurements to estimate morphometric features of different body regions, such as stature and proportional dimensions. These approaches are expected to enhance the evidentiary value of footprints by enabling both the biological profiling and the reconstruction of their anatomical attributes, thus providing a scientifically grounded framework that can support criminal investigations and the broader field of forensic sciences.

## 2. Materials and Methods

### 2.1. Ethical Considerations

This study was approved by the Non-Interventional Clinical Research Ethics Committee of the Faculty of Medicine, Çukurova University (Decision No. 9, Meeting No. 129, dated 6 January 2023). Between September 2023 and February 2024, a total of 100 healthy adult volunteers were prospectively evaluated through foot impression and body measurements.

### 2.2. Participants and Study Design

A total of 100 healthy adult volunteers (*n* (male) = 48, *n* (female) = 52) aged 18–49 years were prospectively recruited. This age range was deliberately chosen to include only young and early middle-aged adults: because foot and footprint dimensions change during growth and maturation, equations derived from adults are not transferable to subadults [11], while restricting the upper age limit minimized potential confounders such as age-related stature reduction and foot-arch flattening. Participants with congenital anomalies or a history of surgical operations that could affect hand, foot, or body dimensions were excluded. To ensure measurement standardization, all measurements were performed by the same researcher. Footprints were obtained by applying charcoal powder on a white background, and each print was photographed with a ruler placed beside it for calibration. Static (standing) bilateral footprints were obtained using a contrast-medium impression technique; in contrast to dynamic prints produced during locomotion, static prints are recorded while the participant stands still [12]. Charcoal powder was selected as the contrast medium because it is readily available, inexpensive, easily removed from the skin, and non-toxic and mostly hypoallergenic, making it well suited for use with human volunteers. After the plantar surface was cleaned, a thin and even layer of fine charcoal powder was applied, and each participant was asked to stand and place the foot on white background under standardized, controlled conditions to obtain a clear static impression. Each print was photographed alongside a scale bar for calibration and subsequently digitized in ImageJ (National Institutes of Health, Bethesda, MD, USA; v.1.54j). As only static prints were analysed, the ghosting phenomenon characteristic of dynamic footprints—which can enlarge length measurements—did not apply to our data [13]. Footprint collection and measurement followed established forensic-podiatry procedures [14,15]. Body measurements were taken using a measuring tape.

### 2.3. Parameters of Body (Figure 1)

The following body parameters were recorded for each participant:

B1—Maximum Cranial Breadth (eu–eu): Also referred to as the maximum transverse diameter, this measurement represents the linear distance between the bilateral euryon (eu) points located on the most lateral aspects of the parietal bones.
B2—Total Facial Height (tr–me): This parameter represents the linear distance between the menton (me)—the lowest point on the chin in the midline—and the trichion (tr), which is the point at the junction of the forehead and the hairline when viewed from the anterior aspect.
B3—Neck Width: Measured as the maximum linear distance across the neck in the coronal plane at the level of the prominentia laryngea (laryngeal prominence), while the subject maintains an upright posture and looks straight ahead.
B4—Neck Height: This measurement represents the linear distance between the menton (me) and the junction of the sternocleidomastoid muscle with the sternum, taken while the subject maintains an upright posture with the neck held straight and the gaze directed forward.
B5—Upper Limb Length: Measured as the linear distance between the tips of the third fingers (digitus medius) of both hands while the subject is in the anatomical position, with the arms relaxed and hanging alongside the trunk.
B6—Arm Span: Defined as the linear distance between the acromion and the tip of the third finger (digitus medius) while the subject is in the anatomical position with the arms relaxed alongside the body.
B7—Biacromial Width: Measured as the linear distance between the acromial extremities of both scapulae while the subject is in the anatomical position with the arms relaxed alongside the trunk.
B8—Thorax Width: Defined as the linear distance between the right and left midaxillary lines at the level of the midline of the chest while the subject is in the anatomical position.
B9—Abdominal Width: Measured as the linear distance between the right and left midaxillary lines at the level of the umbilicus, while the subject is in the anatomical position.
B10—Bicristal Width: Defined as the linear distance between the most lateral points of the right and left iliac crests while the subject is in the anatomical position.
B11—Lower Limb Length: Measured as the linear distance between the anterior superior iliac spine (ASIS) and the medial malleolus while the subject is in the anatomical position.

**Figure 1:**
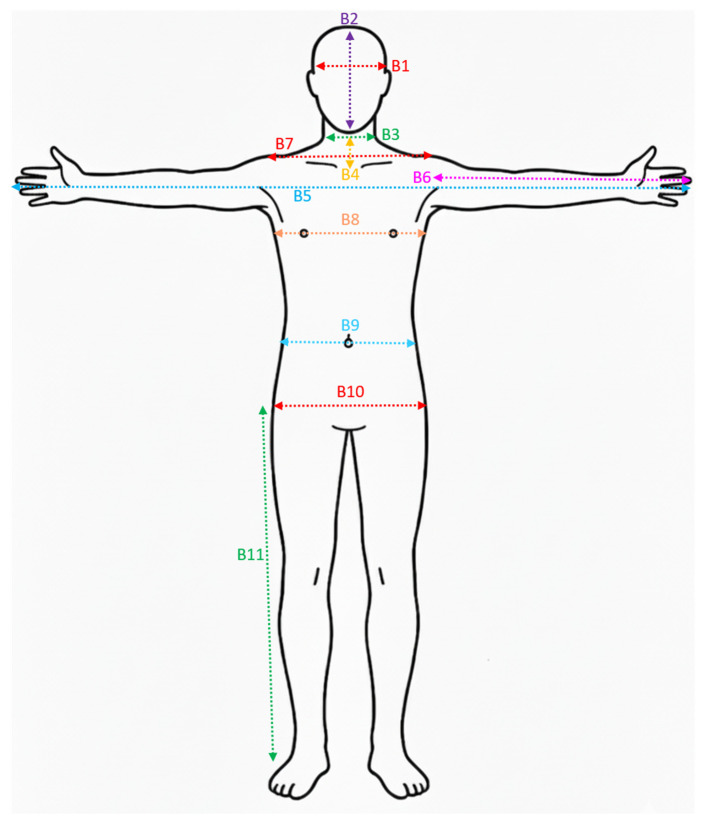
Anthropometric body measurement parameters (B1–B11). B1, maximum cranial breadth (euryon–euryon, eu–eu); B2, total facial height (trichion–menton, tr–me); B3, neck width; B4, neck height; B5, upper limb length; B6, arm span; B7, biacromial width; B8, thorax width; B9, abdominal width; B10, bicristal width; B11, lower limb length. Landmarks and measurement definitions follow standard anthropometric protocols [16].

### 2.4. Parameters of Footprint (Figure 2)

F1—Distance Between the Most Posterior Point of the Heel and the Tip of the First Toe: Defined as the linear distance from the most posterior point of the heel to the tip of the first toe on the footprint.
F2—Distance Between the Most Posterior Point of the Heel and the Tip of the Second Toe: Measured as the linear distance from the most posterior point of the heel to the tip of the second toe on the footprint.
F3—Distance Between the Most Posterior Point of the Heel and the Tip of the Third Toe: Defined as the linear distance from the most posterior point of the heel to the tip of the third toe on the footprint.
F4—Distance Between the Most Posterior Point of the Heel and the Tip of the Fourth Toe: Measured as the linear distance from the most posterior point of the heel to the tip of the fourth toe on the footprint.

**Figure 2:**
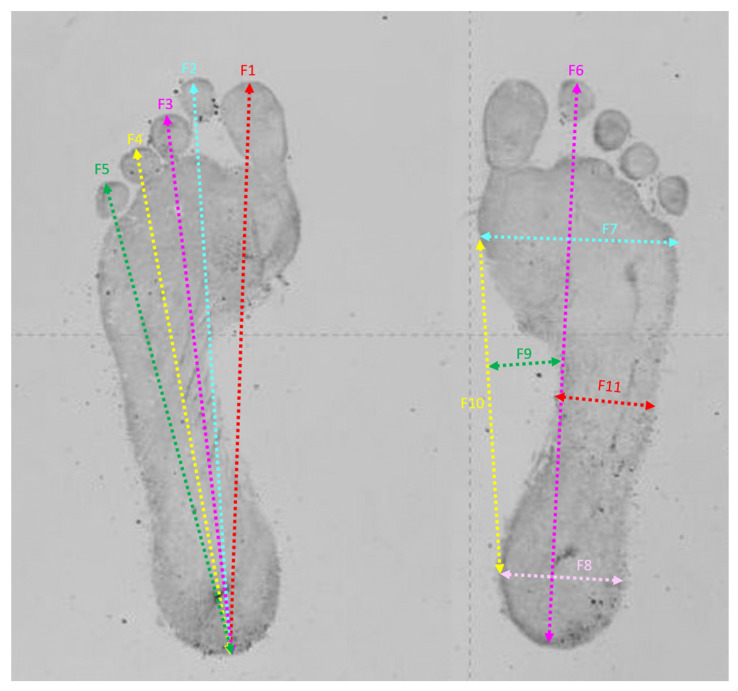
Footprint measurement parameters (F1–F11) obtained from static charcoal-powder prints. F1–F5, distances from the most posterior point of the heel to the tips of the first to fifth toes, respectively; F6, maximum footprint length; F7, maximum footprint width; F8, heel width; F9, medial longitudinal arch width; F10, medial longitudinal arch length; F11, midfoot width. Footprint dimensions were measured following established forensic podiatry protocols [14,17].

F5—Distance Between the Most Posterior Point of the Heel and the Tip of the Fifth Toe: Defined as the linear distance from the most posterior point of the heel to the tip of the fifth toe on the footprint.
F6—Maximum Footprint Length: Measured as the linear distance between the most posterior point of the heel and the most anterior point of the footprint.
F7—Maximum Footprint Width: Defined as the linear distance between the most lateral points of the footprint in the coronal plane.
F8—Heel Width: Measured as the linear distance between the most lateral points of the heel region of the footprint.
F9—Medial Longitudinal Arch Width: Defined as the linear distance from the most medial point of the midfoot to the imaginary line representing the medial longitudinal arch of the footprint.
F10—Medial Longitudinal Arch Length: Measured as the linear distance between the most medial point of the metatarsal region and the most medial point of the heel on the footprint.
F11—Midfoot Width: Measured as the linear distance across the narrowest part of the midfoot region on the footprint.

### 2.5. Statistical Analysis

Descriptive statistics and initial group comparisons were performed using IBM SPSS Statistics, version 29.0.2.0 (IBM Corp., Armonk, NY, USA). Descriptive statistics were presented as mean ± standard deviation, median, minimum–maximum values, and frequency with percentage, as appropriate. The normality of continuous variables was assessed using the Shapiro–Wilk test. Sex-based comparisons were performed using the independent samples t-test for normally distributed variables and the Mann–Whitney U test for non-normally distributed variables. Right and left footprint measurements were compared using paired-samples t-tests or Wilcoxon signed-rank tests, depending on the distribution of the paired differences. Effect sizes for sex-based comparisons were reported using Cohen’s d with 95% confidence intervals.

Additional model-based analyses, including internal validation procedures, multicollinearity diagnostics, and regression model validation, were performed using R statistical software via the RStudio IDE, version 2026.05.1 Build 225 (Posit Software, PBC, Boston, MA, USA). The R packages used for these analyses included MASS for discriminant function analysis, pROC for receiver operating characteristic analysis, car for variance inflation factor diagnostics, and effectsize for effect size estimation. Discriminant function analyses were performed separately for right and left footprint measurements to estimate sex using F1–F11 parameters. Apparent classification accuracy, sex-specific correct classification rates, Wilks’ Lambda, eigenvalues, and group centroids were reported. To evaluate optimism in classification performance, the discriminant models were internally validated using both leave-one-out cross-validation and stratified 10-fold cross-validation. Classification accuracy and 95% confidence intervals were reported separately for apparent and internally validated performance.

Pearson correlation analysis was used to evaluate morphometric associations between footprint and body measurements. Initial exploratory multiple linear regression models were developed using footprint parameters as predictors of stature and body measurements. Because several footprint length variables were highly correlated, multicollinearity was assessed using variance inflation factors. To reduce model instability, reduced morphometric regression models were additionally evaluated using a predefined set of representative footprint parameters: maximum footprint length (F6), maximum footprint width (F7), heel width (F8), medial longitudinal arch length (F10), and midfoot width (F11). Regression model performance was reported using the apparent adjusted R^2^ and standard error of estimate. Internal validation of the reduced regression models was performed using repeated 10-fold cross-validation, and predictive error was quantified using root mean square error, mean absolute error, and cross-validated R^2^. Models showing weak or unstable cross-validated performance were interpreted as exploratory rather than robust predictive equations.

A two-sided *p*-value < 0.05 was considered statistically significant.

### 2.6. Landmark Calibration and Intra-Observer Reliability Assessment

Before formal measurements, all anatomical landmarks were meticulously defined according to a standardized morphometric measurement protocol. The observer underwent intensive training based on these predefined landmark definitions, and landmark placement accuracy was strictly reviewed prior to data collection to ensure methodological consistency. To minimize environmental and measurement-related variability, all digital assessments were performed under identical image analysis conditions. To assess intra-observer reliability, 30 randomly selected images (*n* = 30) were re-measured independently by the same investigator using the same standardized digital measurement protocol. Reliability was evaluated using the intraclass correlation coefficient (ICC) based on a two-way mixed-effects absolute-agreement model. Both single-measure and average-measure ICC values, along with their 95% confidence intervals (CI), were reported. The intra-observer reliability analysis demonstrated excellent reproducibility across all analyzed parameters. The single-measure ICC values ranged from 0.998 (95% CI: 0.995–0.999) to 1.000 (95% CI: 1.000–1.000), while the average-measure ICC values ranged from 0.999 to 1.000. All ICC values were statistically significant (*p* < 0.001), confirming excellent intra-observer reproducibility of the repeated measurements. A *p*-value < 0.05 was considered statistically significant for all analyses.

## 3. Results

Among the 100 adult volunteers (min: 18, max: 49) included in the study, 52 were female (52%) and 48 were male (48%). The mean age ± standard deviation (SD) was 36.25 ± 15.60 years for females and 35.81 ± 14.08 years for males. The mean height ± SD of the participants was 163.94 ± 5.25 cm for females and 177.79 ± 7.37 cm for males. The mean body weight ± SD was 66.84 ± 17.86 kg for females and 81.62 ± 13.31 kg for males.

Table 1 presents the descriptive statistics for 11 different body measurements obtained from male (*n* = 48) and female (*n* = 52) participants, along with *p*-values indicating the statistical significance of sex differences. In all variables except bicristal width (B10), males exhibited significantly higher mean values than females (*p* < 0.05, Table 1). The most pronounced differences were observed in upper limb length (B5), arm span (B6), and lower limb length (B11), which serve as major indicators of overall body size. Only bicristal width did not show a statistically significant sex difference (*p* = 0.515). These findings highlight a clear and consistent sexual dimorphism in somatic dimensions between the sexes.

Table 2 summarizes the 11 right footprint anthropometric measurements and compares them between males (*n* = 48) and females (*n* = 52). For all assessed parameters, males showed significantly larger measurements compared to females (*p* < 0.05). Particularly, footprint length (F6), footprint width (F7), and heel-to-toe distances (F1–F5) exhibited highly significant sex-based differences (*p* < 0.001). These results demonstrate robust sexual dimorphism in foot morphology, likely reflecting overall skeletal size variations alongside potential functional adaptations.

Table 3 displays the left footprint measurements analogous to those presented in Table 2. Consistent with the right foot findings, all variables except midfoot width (F11; *p* = 0.216) were significantly greater in males than in females (*p* < 0.05). The medial longitudinal arch length (F10) and maximum footprint length (F6) showed particularly strong statistical differences (*p* < 0.001). These data confirm the bilateral symmetry of sexual dimorphism in human footprint dimensions, demonstrating their practical utility for forensic, ergonomic, and anthropometric applications.

### 3.1. Discriminant Function Analysis of Right Footprint Measurements

A multivariate discriminant function was constructed using eleven right-foot measurement parameters (F1–F11) to differentiate between male and female participants. The resulting discriminant equation was as follows:

D = −5.978 + (0.143 × F1) + (0.165 × F2) + (0.025 × F3) + (0.014 × F4) − (0.064 × F5) − (0.252 × F6) − (0.016 × F7) − (0.057 × F8) + (0.029 × F9) − (0.004 × F10) + (0.021 × F11)

The model showed a Wilk’s Lambda value of 0.763 and an eigenvalue of 0.311, indicating a moderate level of discriminatory power. The group centroid values were 0.574 for males and −0.530 for females, demonstrating distinct group means for the two sexes. The canonical discriminant function successfully classified 74% of the overall cases correctly. The model demonstrated a higher classification accuracy rate for females (80.8%, *n* = 42) compared to males (66.7%, *n* = 32).

### 3.2. Discriminant Function Analysis of Left Footprint Measurements

A multivariate discriminant function was generated using eleven left-foot measurement parameters (F1–F11) to classify individuals by sex. The resulting discriminant equation was:

D = −5.978 + (0.049 × F1) +(0.086 × F2) + (0.061 × F3) − (0.028 × F4) − (0.096 × F5) − (0.052 × F6) − (0.043 × F7) − (0.019 × F8) − (0.019 × F9) + (0.030 × F10) − (0.013 × F11)

The model yielded a Wilk’s Lambda value of 0.771 and an eigenvalue of 0.296, indicating a moderate level of discriminatory ability. Group centroid values were 0.561 for males and −0.518 for females, suggesting distinguishable distributions between the two sexes. The function correctly classified 71% of the total cases, with a higher accuracy rate in females (76.9%, *n* = 40) compared to males (64.6%, *n* = 31).

For the right-footprint model, the apparent classification accuracy was 74.0% (95% CI: 64.3–82.3%). Internal validation reduced classification accuracy to 65.0% (95% CI: 54.8–74.3%) with leave-one-out cross-validation and to 65.0% (95% CI: 54.8–74.3%) with stratified 10-fold cross-validation. For the left-footprint model, the apparent classification accuracy was 71.0% (95% CI: 61.1–79.6%), whereas the corresponding internally validated accuracies were 64.0% (95% CI: 53.8–73.4%) and 67.0% (95% CI: 56.9–76.1%), respectively.

### 3.3. Regression-Based Estimation of Stature and Arm Span from Reduced Footprint Models

Because the initial regression models included multiple highly correlated footprint length variables, multicollinearity diagnostics indicated substantial collinearity among predictors in the full exploratory models; these diagnostics are provided in Supplementary Tables S1 and S2. Therefore, in addition to the full exploratory models, reduced morphometric regression models were evaluated using a predefined set of representative footprint parameters: maximum footprint length (F6), maximum footprint width (F7), heel width (F8), medial longitudinal arch length (F10), and midfoot width (F11). These reduced models were internally validated using repeated 10-fold cross-validation.

In the reduced morphometric regression models, the most stable validated performance was observed for stature and arm span (Table 4). For stature estimation, the apparent adjusted R^2^ values were 0.275 for the right foot and 0.268 for the left foot, whereas the corresponding cross-validated R^2^ values were 0.200 and 0.174, respectively. For arm span estimation, the apparent adjusted R^2^ was 0.303 for both feet, with cross-validated R^2^ values of 0.230 for the right foot and 0.219 for the left foot. These findings indicate weak but relatively stable validated predictive performance. In contrast, the remaining body-measurement models showed very weak or unstable cross-validated performance and were therefore interpreted as exploratory rather than as robust predictive equations. Full exploratory regression equations for all body measurements, together with multicollinearity diagnostics and internal validation metrics, are provided in Supplementary Tables S1 and S2.

## 4. Discussion

Forensic anthropology plays a crucial role in legally mandated identification processes, particularly in cases involving severe body damage, fragmented remains, or mass fatality events. By contributing to the reconstruction of biological profiles, forensic anthropological methods may help narrow the pool of possible identities when primary identifiers are unavailable or compromised [18]. Within this context, footprints represent a potentially useful form of physical evidence because they reflect both individual plantar morphology and broader somatic characteristics.

Footprints have long been considered valuable in forensic investigations because the human foot produces measurable and partially individualized impressions, excluding dermatoglyphic patterns [19]. In the present study, static footprint morphometry demonstrated clear sex-related differences in most measured footprint parameters. These findings support the view that footprint morphology contains sexually dimorphic information that may contribute to biological profiling. However, the practical interpretation of such evidence should remain cautious, particularly because the present models were developed from standardized static footprints obtained under controlled conditions rather than from partial, distorted, or substrate-dependent impressions typically encountered in real crime-scene settings.

Sex estimation in forensic anthropology traditionally relies on highly dimorphic skeletal structures, particularly the pelvis and skull. The pelvis is considered the most reliable skeletal region for sex estimation when sufficiently preserved, whereas cranial and mandibular parameters may provide additional information in incomplete remains [20,21,22]. Previous studies have reported high sex estimation accuracies using pelvic and selected craniofacial or postcranial measurements [23,24,25]. In comparison, footprint-based approaches generally provide supportive rather than definitive evidence, and their accuracy varies depending on population characteristics, measurement technique, and model validation strategy.

Several previous studies have reported higher sex estimation accuracy using direct foot measurements or more advanced computational methods. Zeybek et al. [24] reported sex prediction accuracies exceeding 95% using foot length, width, and height measurements in a Turkish sample. Similarly, Parlak et al. [26] reported accuracies of 94.0% for the right foot and 94.8% for the left foot using multiple discriminant analysis, with further improvement when artificial neural networks were applied. Other footprint-based studies have reported more moderate accuracies. Suleiman et al. [27] identified left foot width and right foot length as the best single predictors, with accuracies of 72.5% and 71.7%, respectively, while Abledu et al. [28] reported univariate footprint-based accuracies ranging from 69.8% to 80.3%. The apparent classification accuracies observed in the present study, 74.0% for the right footprint model and 71.0% for the left footprint model, are therefore within the range reported for footprint-based sex estimation, but lower than those achieved using direct foot measurements or advanced modelling approaches.

Importantly, the present study further evaluated the stability of the discriminant models using internal validation. After leave-one-out and stratified 10-fold cross-validation, sex classification performance decreased to approximately 64–67%. This decline indicates that the apparent classification accuracy was partly optimistic and that the validated discriminative performance should be interpreted as moderate. Thus, although static footprint morphology appears to contain meaningful sexually dimorphic information, the resulting discriminant models should be regarded as ancillary tools rather than standalone methods for forensic sex estimation. The higher classification accuracy observed among females in both right- and left-footprint models may reflect sex-specific differences in the distribution of footprint dimensions within this sample, but this finding requires confirmation in larger and independent datasets.

The observed sexual dimorphism in footprint morphology is biologically plausible. Males generally exhibit greater skeletal dimensions, bone robusticity, and muscle mass as a result of sex-related differences in growth, maturation, and hormonal influences. These differences are reflected in foot size and shape, leading to larger footprint dimensions in males. In the present study, most footprint parameters were significantly larger in males, supporting the presence of measurable sex-related variation in static footprints. Nevertheless, the moderate validated classification performance indicates that overlap between male and female footprint measurements remains substantial.

Beyond sex estimation, footprint analysis has most commonly been investigated for stature estimation. Previous studies have shown that footprint length-related measurements tend to be more strongly associated with stature than width-related parameters. Krishan [14] reported relatively low mean errors for stature estimation using heel-to-toe footprint measurements, and comparable findings have been reported in other populations [29,30,31,32]. In a Slovak adult sample, stature was predicted from static footprints with an error of approximately 4.4 cm; however, the authors emphasized that such equations are population-specific and should not be generalized without validation [12]. These findings are consistent with the broader anthropometric principle that longitudinal dimensions of the foot are related to overall body height.

In the present study, the initial exploratory regression models suggested apparent associations between footprint parameters and several body measurements. However, multicollinearity diagnostics indicated substantial collinearity among length-related footprint variables, particularly because several heel-to-toe distances and maximum footprint length captured overlapping morphometric information. To address this issue, reduced morphometric regression models were evaluated using representative footprint parameters: maximum footprint length, maximum footprint width, heel width, medial longitudinal arch length, and midfoot width. These reduced models were internally validated using repeated 10-fold cross-validation.

After internal validation, the most stable regression performance was observed for stature and arm span. For stature estimation, the cross-validated R^2^ values were 0.200 for the right foot and 0.174 for the left foot. For arm span estimation, the corresponding cross-validated R^2^ values were 0.230 and 0.219, respectively. These values indicate weak but relatively stable validated predictive performance. In contrast, the remaining body measurement models showed very weak or unstable cross-validated performance, with several models performing worse than prediction based on the sample mean. Therefore, the regression equations should be interpreted as exploratory and supportive rather than robust predictive tools for body proportion reconstruction.

The concept of body proportion, or “canon,” has historical relevance in both artistic anatomy and anthropometry [9]. In forensic contexts, selected body dimensions such as stature, arm span, limb length, shoulder breadth, thoracic width, and pelvic width may contribute to the characterization of body build. However, the present findings suggest that although some of these dimensions are statistically associated with footprint morphology, the validated predictive performance is limited. Consequently, footprint-derived estimates of somatic proportions should not be used as independent evidence for individual identification. Rather, they may provide Supplementary Information when interpreted together with other forensic, anthropological, or circumstantial evidence.

The population-specific nature of footprint morphology should also be emphasized. Footprint dimensions may vary according to genetic background, environmental factors, nutrition, lifestyle, footwear habits, occupation, and age-related musculoskeletal changes. Therefore, prediction equations developed in one population may not be directly applicable to another. The present study contributes data from a Turkish adult sample, but external validation in independent Turkish and non-Turkish populations is required before the models can be recommended for broader forensic use.

Overall, the present findings support the potential value of static footprint morphometry as a supportive source of information for sex estimation and selected somatic dimensions, particularly stature and arm span. However, the internally validated results also show that the predictive capacity of the models is modest. Accordingly, the forensic applicability of these models should be framed conservatively: they may assist biological profiling in standardized conditions but should not replace primary identification methods or more established anthropological indicators.

## 5. Limitations

This study has several limitations. First, the sample consisted of healthy adults from a specific Turkish population; therefore, the developed models may not be directly applicable to populations with different anthropometric characteristics. Since footprint morphology may be influenced by genetic, environmental, nutritional, lifestyle-related, and occupational factors, population-specific differences should be considered when interpreting or applying these models. In addition, no a priori sample size calculation was performed; therefore, the sample size should be considered a limitation, particularly given the number of predictors used in the exploratory models.

Second, the study included a broad adult age range. Although all participants were adults, potential age-related changes in stature, soft-tissue distribution, foot structure, arch morphology, and body proportions were not evaluated separately. Such changes may influence footprint morphology and may affect the predictive performance of both discriminant and regression models. Future studies should examine age-stratified models or include age-adjusted prediction approaches.

Third, footprint acquisition was limited to static barefoot footprints obtained under standardized laboratory conditions. Real forensic casework often involves partial, distorted, dynamic, or substrate-dependent impressions. Environmental conditions, surface characteristics, pressure distribution, movement dynamics, and post-depositional changes may all influence footprint morphology and measurement accuracy. Therefore, the present findings are most directly applicable to standardized static footprints and may not fully reflect performance under non-ideal forensic conditions.

Fourth, charcoal powder impressions were used as the contrast medium. Although this method is inexpensive, practical, non-toxic, and suitable for standardized volunteer-based data collection, it has not been formally validated against ink-based, digital, or three-dimensional footprint acquisition methods. Potential systematic measurement differences between acquisition techniques cannot be excluded.

Fifth, all measurements were performed by a single trained observer. Although intra-observer reliability was excellent, inter-observer reliability could not be assessed. This is an important limitation for forensic applications, where evidence may be evaluated by different practitioners. Future studies should include multiple observers and assess both intra- and inter-observer reproducibility.

Sixth, although the discriminant and regression models were internally validated using cross-validation, they were not externally validated in an independent sample. Therefore, the reported performance should still be interpreted cautiously. External validation in larger, independent, and more diverse populations is required to assess generalizability and reproducibility.

Finally, several footprint predictors were highly correlated, particularly length-related measurements. Although reduced morphometric models were used to decrease multicollinearity and improve model stability, the validated regression performance remained weak for most body measurements. Therefore, the regression equations should be considered exploratory and should not be used as standalone predictive tools in forensic practice.

## 6. Conclusions

This study demonstrates that static footprint morphometry contains measurable sexually dimorphic information in a Turkish adult sample. The apparent discriminant classification accuracies were 74.0% for the right foot and 71.0% for the left foot; however, internal validation reduced the classification performance to approximately 64–67%, indicating moderate discriminative ability. These findings suggest that footprint measurements may provide ancillary information for sex estimation, but they should not be regarded as standalone forensic identification tools.

Regression analyses showed that reduced footprint models provided the most stable validated performance for stature and arm span estimation. However, the cross-validated R^2^ values remained weak, and most other body measurement models showed very weak or unstable validated performance. Therefore, footprint-based reconstruction of body proportions should be interpreted as exploratory and supportive rather than definitive.

In forensic practice, static footprint morphometry may contribute to biological profiling when interpreted together with other evidence, particularly in situations where primary identifiers are unavailable or compromised. Nevertheless, the proposed models require external validation in larger and more diverse populations, assessment under realistic forensic conditions, and evaluation of inter-observer reliability before routine application can be recommended. Future research should also compare different footprint acquisition techniques, examine dynamic and partial footprints, and explore validated modelling approaches that can improve predictive stability without overstating forensic applicability.

## Figures and Tables

**Table 1:** Descriptive statistics and sex-based comparisons of body measurements (B1–B11) in male and female participants (mm).

Parameters	Mean ± SD (Male)	Mean ± SD (Female)	Cohen’s d (95% CI)	*p*
B1—Maximum Cranial Breadth (eu–eu)	173.85 ± 12.51	157.88 ± 13.55	1.228 (0.925–1.530)	<0.001
B2—Total Facial Height (tr–me)	191.04 ± 17.47	177.98 ± 14.59	0.817 (0.527–1.105)	<0.001
B3—Neck Width	116 ± 10.18	104.81 ± 12.58	0.998 (0.702–1.291)	<0.001
B4—Neck Height	110.31 ± 12.69	99.13 ± 10.61	0.964 (0.670–1.256)	<0.001
B5—Upper Limb Length	725.72 ± 40.19	676.53 ± 41.24	1.213 (0.910–1.514)	<0.001
B6—Arm Span	1781.66 ± 88.27	1643.65 ± 78.62	1.660 (1.336–1.980)	<0.001
B7—Biacromial Width	428.364 ± 27.18	375.03 ± 28.12	1.917 (1.580–2.250)	<0.001
B8—Thorax Width	346.15 ± 27.42	305.57 ± 36.41	1.256 (0.951–1.558)	<0.001
B9—Abdominal Width	323.33 ± 22.69	281.44 ± 47.02	1.126 (0.826–1.424)	<0.001
B10—Bicristal Width	362.29 ± 27.89	359.04 ± 41.71	0.091 (−0.187–0.368)	0.515
B11—Lower Limb Length	1002.08 ± 63.78	942.69 ± 51.09	1.032 (0.736–1.327)	<0.001

**Table 2:** Descriptive statistics and sex-based comparisons of right footprint measurements (F1–F11) (mm).

Parameters	Mean ± SD Male	Mean ± SD Female	Cohen’s d (95% CI)	*p*
F1—Distance Between the Most Posterior Point of the Heel and the Tip of the First Toe	262.40 ± 37.21	236.17 ± 23.33	0.852 (0.442–1.260)	<0.001
F2—Distance Between the Most Posterior Point of the Heel and the Tip of the Second Toe	262.90 ± 38.23	234.99 ± 23.88	0.883 (0.470–1.293)	<0.001
F3—Distance Between the Most Posterior Point of the Heel and the Tip of the Third Toe	251.76 ± 37.11	225.33 ± 22.69	0.867 (0.455–1.276)	<0.001
F4—Distance Between the Most Posterior Point of the Heel and the Tip of the Fourth Toe	238.84 ± 34.66	214.55 ± 21.64	0.848 (0.436–1.256)	<0.001
F5—Distance Between the Most Posterior Point of the Heel and the Tip of the Fifth Toe	221.57 ± 31.71	200.48 ± 20.34	0.798 (0.389–1.204)	<0.001
F6—Maximum Footprint Length	263.97 ± 14.69	237.71 ± 23.80	0.838 (0.426–1.245)	<0.001
F7—Maximum Footprint Width	96.91 ± 14.69	87.80 ± 9.25	0.749 (0.341–1.153)	<0.001
F8—Heel Width	58.27 ± 10.48	53.01 ± 5.42	0.639 (0.235–1.039)	0.003
F9—Medial Longitudinal Arch Width	39.87 ± 9.19	36.01 ± 5.76	0.507 (0.107–0.904)	0.013
F10—Medial Longitudinal Arch Length	125.17 ± 20.45	114.73 ± 14.60	0.591 (0.189–0.991)	0.005
F11—Midfoot Width	37.87 ± 8.90	34.25 ± 7.93	0.431 (0.033–0.827)	0.035

**Table 3:** Descriptive statistics and sex-based comparisons of left footprint measurements (F1–F11) (mm).

Parameters	Mean ± SD Male	Mean ± SD Female	Cohen’s d (95% CI)	*p*
F1—Distance Between the Most Posterior Point of the Heel and the Tip of the First Toe	262.58 ± 37.95	236.46 ± 22.06	0.850 (0.438–1.258)	<0.001
F2—Distance Between the Most Posterior Point of the Heel and the Tip of the Second Toe	261.75 ± 38.85	234.39 ± 22.76	0.868 (0.455–1.276)	<0.001
F3—Distance Between the Most Posterior Point of the Heel and the Tip of the Third Toe	250.92 ± 37.24	225.04 ± 22.70	0.847 (0.435–1.255)	<0.001
F4—Distance Between the Most Posterior Point of the Heel and the Tip of the Fourth Toe	237.51 ± 35.58	214.01 ± 21.14	0.811 (0.401–1.218)	<0.001
F5—Distance Between the Most Posterior Point of the Heel and the Tip of the Fifth Toe	220.72 ± 32.63	199.67 ± 19.61	0.789 (0.380–1.195)	<0.001
F6—Maximum Footprint Length	263.98 ± 38.08	237.72 ± 22.15	0.852 (0.440–1.260)	<0.001
F7—Maximum Footprint Width	96.82 ± 14.46	88.86 ± 8.45	0.678 (0.273–1.080)	0.001
F8—Heel Width	57.66 ± 10.55	52.63 ± 6.55	0.577 (0.175–0.976)	0.005
F9—Medial Longitudinal Arch Width	38.58 ± 9.23	35.20 ± 6.19	0.433 (0.035–0.829)	0.036
F10—Medial Longitudinal Arch Length	123.88 ± 20.91	109.80 ± 14.73	0.784 (0.374–1.189)	<0.001
F11—Midfoot Width	37.49 ± 9.08	35.41 ± 7.63	0.249 (−0.145–0.642)	0.216

**Table 4:** Internally validated regression performance of reduced footprint models for stature and arm span estimation.

Foot	Outcome	Apparent Adj. R^2^	SEE	CV-RMSE	CV-MAE	CV-R^2^
Right	Stature	0.275	80.07	83.70	65.21	0.200
Right	Arm span	0.303	90.35	94.50	73.47	0.230
Left	Stature	0.268	80.45	85.02	65.81	0.174
Left	Arm span	0.303	90.36	95.18	73.49	0.219

Reduced morphometric models included F6, F7, F8, F10, and F11 as predictors. Apparent adjusted R^2^ and SEE were derived from the full dataset, whereas CV-RMSE, CV-MAE, and CV-R^2^ were obtained using repeated 10-fold cross-validation. SEE, CV-RMSE, and CV-MAE are expressed in millimeters. CV, cross-validation; RMSE, root mean square error; MAE, mean absolute error; SEE, standard error of estimate.

## Data Availability

The original contributions presented in this study are included in the article. Further inquiries can be directed to the corresponding author.

## Supplementary Materials

The following supporting information can be downloaded at https://www.mdpi.com/article/10.3390/diagnostics16132114/s1, Table S1: Exploratory regression models for right footprint parameters; Table S2: Exploratory regression models for left footprint parameters.
