# Supplementary material for: Footprints as Morphometric Evidence for Somatic Prediction and Body Proportion Reconstruction in Forensic Medicine
Source: Diagnostics (Basel). 2026 Jul 6;16(13):2114. doi: 10.3390/diagnostics16132114 (PMC13362048; doi:10.3390/diagnostics16132114)
Supplement: Supplementary file 1 [file diagnostics-16-02114-s001.zip › diagnostics-4365546-supplementary.pdf]

Supplementary

Table S1: Exploratory regression models for right footprint parameters

Equations	Adjusted R^2	SEE	p	Max VIF	CV-RMSE	CV-MAE	CV-R^2
Stature= 1958.924 – (12.234 x F1) – (4.776 x F5) + (14.864 x F6) + (2.629 x F7) – (2.571 x F9)	0.269	63.02	0.002	164.06	83.91	64.77	0.195
B1- Maximum Cranial Breadth (eu–eu)= 144.765 + (0.671 x F1) + (0.853 x F3) – (0.657 x F5) – (0.897 x F6) + (0.378 x F11)	0.099	14.50	0.011	205.36	14.94	11.08	0.034
B2- Total Facial Height (tr–me)= 163.053 – (0.404 x F5) + (0.460 x F6) – (0.674 x F8) + (0.433 x F9) + (0.343 x F11)	0.033	16.99	0.149	33.14	17.75	14	-0.065
B3- Neck Width = 99.030 + (0.710 x F1) + (0.416 x F2) – (1.099 x F6) + (0.179 x F11)	0.011	12.75	0.288	313.73	13.15	10.33	-0.061
B4- Neck Height= 90.271 + (0.515 x F1) + (1.254 x F2) – (0.490 x F4) – (1.254 x F6) – (0.382 x F7) + (0.479 x F8) + (0.217 x F9)	0.095	12.25	0.022	318.66	13.11	10.28	-0.047
B5- Upper Limb Length= 578.237 + (2.347 x F2) – (1.838 x F5) – (1.036 x F9) – (0.961 x F11)	0.215	42.07	<0.001	32.07	43.35	34.31	0.158
B6- Arm Span = 1263.011 + (6.018 x F2) – (4.083 x F3) – (2.009 x F9)	0.353	87.03	<0.001	74.23	89.52	70.37	0.309
B7- Biacromial Width = 311.266 + (2.858 x F2) – (1.672 x F5) – (1.464 x F6) + (0.788 x F7) + (0.725 x F11)	0.213	33.89	<0.001	115.4	35.2	28.68	0.143
B8- Thorax Width = 347.403 + (1.212 x F1) – (1.568 x F5) – (2.444 x F8) + (1.419 x F9) + (2.367 x F11)	0.114	45.05	0.005	26.15	38.56	31.37	-0.03
B9- Abdominal Width= 263.270 + (0.979 x F1) – (1.600 x F5) + (1.879 x F9) + (1.668 x F11)	0.075	41.10	0.022	25.68	42.39	35.02	0.006
B10- Bicristal Width= 321.640 – (2.254 x F2) + (2.227 x F6) + (0.802 x F11)	0.055	34.79	0.038	104.96	36.08	26.04	-0.026
B11- Lower Limb Length= 914.177 + (2.085 x F1) + (5.577 x F4) – (5.602 x F5) – (3.433 x F7) – (1.972 x F10)	0.100	102.95	0.010	68.82	61.51	45.52	0.09

Table S2: Exploratory regression models for left footprint parameters

Equations	Adjusted R^2	SEE	p	Max VIF	CV-RMSE	CV-MAE	CV-R^2
Stature =1329.910 + (2.298 x F1) + (2.294 x F2) – (3.641 x F5)	0.317	77.71	<0.001	67.44	80.73	62.21	0.255
B1- Maximum Cranial Breadth (eu–eu)= 142.547 + (0.746 x F3) – (0.804 x F5) + (0.395 x F11)	0.104	14.57	0.004	51.26	14.81	10.95	0.05
B2- Total Facial Height (tr–me)= 154.047 + (0.612 x F1) – (0.673 x F4) + (0.567 x F9) – (0.199 x F10) + (0.871 x F11)	0.126	16.15	0.003	36.65	16.97	13.35	0.026
B3- Neck Width= 95.693 + (0.389 x F1) – (0.396 x F4) – (0.311 x F9) + (0.157 x F10)	0.046	12.52	0.074	36.65	12.79	9.88	-0.004
B4- Neck Height= 92.007 + (0.706 x F2) – (0.611 x F6) – (0.254 x F11)	0.077	12.37	0.013	78.16	12.92	10.05	-0.016
B5- Upper Limb Length= 564.230 + (2.212 x F2) – (1.692 x F5)	0.208	42.23	<0.001	36.42	43.57	34.31	0.149
B6- Arm Span= 1251.143 + (2.060 x F1) + (2.224 x F2) – (2.2883 x F5)	0.334	88.30	<0.001	67.44	91.69	71.74	0.275
B7- Biacromial Width= 295.358 + (1.292 x F2) – (1.127 x F5) + (0.620 x F11)	0.159	35.05	<0.001	36.5	36.14	29.89	0.097
B8- Thorax Width= 319.423 – (2.255 x F4) + (2.072 x F6) – (1.530 x F8) + (2.091 x F11)	0.146	44.23	<0.001	39.62	37.86	30.4	0.007
B9- Abdominal Width= 249.571 – (1.936 x F4) + (1.840 x F6) + (0.754 x F11)	0.050	41.65	0.048	39.02	42.48	35.97	0.001
B10- Bicristal Width= 318.950 + (3.273 x F1) – (1.339 x F4) – (2.024 x F6) + (0.962 x F11)	0.070	34.52	0.028	276.66	35.33	25.65	0.016
B11- Lower Limb Length= 887.096 + (2.455 x F1) + (4.570 x F3) – (5.804 x F4) – (2.366 x F9) – (0.903 x F10) – (3.336 x F11)	0.040	106.29	0.131	110.55	64.31	46.12	0.005
